# The “Purse-string” Technique for Shoulder Stabilization, Description of the Technique, Long term Results and Literature Review

**DOI:** 10.2174/1874325001711010183

**Published:** 2017-02-28

**Authors:** Georgios Arealis, Joana Bento Rodrigues, Natalie Hope, Ofer Levy

**Affiliations:** 1 Reading Shoulder Unit, Royal Berkshire and Berkshire Independent Hospitals, Swallows Croft, Wensley Road, Coley Park, Reading, RG1 6UZ, United Kingdom; 2 Reading Shoulder Unit, Royal Berkshire and Berkshire Independent Hospitals, Reading, UK

**Keywords:** “purse-string” technique, Arthroscopic stabilization, Bankart, Number of anchors, Repair of labrum, Anterior shoulder instability, One anchor

## Abstract

**Background:**

Over the last 2 decades arthroscopic stabilization and Bankart repair has gained popularity due to the advances in materials and surgical techniques. Results of arthroscopic stabilization have been similar to open without the risks of it. The number of anchors used has been suggested to be very important in “spot-weld” arthroscopic stabilization however the “purse-string” technique (PST) can achieve similar results using only one anchor.

We describe technique and long term results from using the PST and search the literature for other papers regarding PST.

**Methods:**

Between 2003 and 2013 a total of 193 patients were operated. Patients included those with anterior instability. Using PubMed relevant studies reporting results of PST were identified.

**Results:**

Mean follow up was 2 (range 0.5 to 3) years. 9 (4.7%) patients experienced recurrent instability. Almost all patients (97%) returned to their sporting and leisure activities and all professional athletes went back to the same sport. One more UK centre reported 6.1% recurrence in 114 patients at 4 years follow up. These results are similar to the published 11% recurrence of instability after “spot-weld” arthroscopic techniques at 11 years clinical follow-up.

**Conclusion:**

This study indicates that PST is safe and effective alternative method for the treatment of anterior shoulder instability. In this technique with one anchor simultaneous repair of labrum, creation of an anterior bumper and capsular shift can be achieved. It has the advantage of being cheaper, faster yet efficient with good long term results and leaves space for revision anchors in case of recurrence.

## INTRODUCTION

Repair of avulsion of the anterior inferior labrum from the glenoid rim that was first described in 1920 by Bankart [1] and earlier by Perthes [2] can be either open or arthroscopic. Over the recent years arthroscopic repair has increasingly popular and results have been very promising especially regarding post-operative stiffness and pain compared to open repair [3]. Patients also tend to prefer arthroscopic over open surgery [4].

Traditionally arthroscopic repair is performed using multiple suture anchors and a minimum of 3 anchors is perceived to be necessary to achieve optimum results restoring the labral “bumper”. [5, 6] The idea of using a single anchor and adapt the open vertical apical suture technique described by S. Copeland [7] in the form of “purse- string” technique was first described in 2006 by O. Levy [8] and was found to be at least as successful as multiple anchor techniques [9]. Since then one more centre has published results using the “purse- string” technique to achieve successful arthroscopic shoulder stabilization [10].

The purpose of this study is to review the recent literature and report the results of Reading Shoulder Unit regarding the arthroscopic “purse- string” technique.

## MATERIALS AND METHOD

### Surgical Technique [8]

The patient is positioned in the beach chair position under general anaesthesia. The lateral decubitus position may be used, although we have found the beach chair position easier as it helps to bring the anteroinferior capsule superiorly and is useful as well in terms of ease of conversion to an open procedure. Examination under anaesthesia should be performed with side-to-side comparisons, and range of motion and the degree and direction of humeral head translation are documented according to the method of Cofield *et al*. [11] The shoulder is prepared for surgery and draped in a sterile manner, and the bony landmarks are marked. The arm is positioned by using longitudinal traction of 8 to 10 lbs, in 30- to 40- of flexion. Lateral traction is applied by the assistant or by placing a kidney dish in the axilla.

The joint is entered with a standard 30- arthroscope from the posterior portal. An initial visualization is performed by insufflating 60 mL of air into the joint using a 20-mL syringe. This allows a quick assessment of the structures before pressurized fluid is introduced to the joint. An anterior portal is next established using an outside-in technique. An 18-gauge spinal needle is passed superior to subscapularis, and the needle is used to check that instruments will be easily able to reach the level of the anteroinferior labrum (6-o’clock position).

When this has been confirmed a definitive portal is established using an 8-mm commercially available clear cannula system. The arthroscopic pump is connected, and a systematic evaluation of the glenohumeral joint is performed to assess anterior labral detachment, capsular tears, superior labral and biceps anchor detachment, bony injuries including bony Bankart and Hill-Sacks lesions, rotator cuff pathology, and the inferior pouch to exclude a Humeral Avulsion of Glenohumeral Ligament lesion. It is important to also view from the anterior portal to assess bony loss adequately. Having confirmed the presence of a Bankart lesion, the next step is meticulous mobilization of the capsulolabral complex off the glenoid neck using an arthroscopic elevator. An Angled liberator is used to release the labrum at the 5- and 6-o’clock positions. A grasper or suture manipulator is used to assess the degree of release which is continued until satisfactory. Angled arthroscopic rasps and a 4.0-mm mechanical shaver are used to gently decorticate the anterior neck being mindful not to exacerbate any bony loss. It is useful to clip the shaver suction tubing at this stage to prevent accidental damage to the capsulolabral tissue. The next step is the placement of a suture anchor in the 4-o’clock position. We have used a bioabsorbable anchor with a polydioxanone suture material. The anchor is positioned at 45- to the frontal plane on the glenoid face on the edge of the cartilage surface. The main difference between standard techniques and the purse-string method is the technique of suture passage through both superior and inferior capsulolabral tissue from approximately 2- to 6-o’clock positions. The inferior suture limb is passed through the capsule at the 6-o’clock position Fig. (**1**). A penetrating grasper is passed through the capsule at the 6-o’clock position, and the suture is delivered into its jaws using a knot pusher. The knot pusher is also passed through the same anterior working portal. We have developed a penetrating grasper specifically for this application, the Sixter penetrating grasper (TAG Medical Products, Kibbutz Gaaton, Israel/ DepuyMitek, Warsaw, USA). The size of the capsule bite will ultimately determine the degree of capsular shift achieved. The superior suture limb is positioned at the 2-o’clock position, and the suture ends are tied. Once the suture is tied, the labrum forms a rolled free edge, and the anterior structures are snugged down onto the convex decorticated surface of the anterior glenoid neck. The capsule is drawn up from a south-to-north position addressing the laxity and restoring the Inferior Glenohumeral Ligament. The position of the anchor at 4-o’clock secures the capsular shift from south to north. As the glenoid is an elliptic-circular structure, applying an apical contracting suture in one area around the glenoid will create a circumferential capsular shift like a ‘purse-string` mechanism. Using this ‘purse-string technique,` a large surface area apposition between the capsule and the glenoid neck is created (and not only ‘spot welding`), as well as an anterior bumper with the mass of gathered tissue within the ‘purse-string` suture Fig. (**2**). Once the purse-string suture has been tied, the resultant “bumper^ of capsulolabral tissue is probed to ensure it is firmly fixed to the glenoid. A rotator interval closure may be performed at this stage if necessary. If necessary, an additional anchor may be placed further superior on the glenoid to supplement the repair.

### Postoperative Managment [8]

Postoperatively, the patient is immobilized in a sling with a body belt for 3 weeks with no external rotation permitted. At 3 weeks, the body belt is removed and pendulum exercises can commence. Formal mobilization from the sling occurs at 6 weeks when physiotherapy commences. Physiotherapy includes mobilization, proprioception rehabilitation, scapular stability, and rotator cuff strengthening. It is recommended that patients do not return to contact or overhead sports for 6 months.

### Patients

The senior author has been using the purse-string technique for arthroscopic stabilization for many years. It has the advantage of being technically simpler, faster and cheaper, avoiding multiple suture anchors.

Between 2003 and 2013 a total of 193 patients were operated with median age 27 (range 15- 74) years old. Of them 142 (73.5%) were male and 3 (1.5%) had bilateral shoulder instability. The patient group included only those with anterior instability with a distinct history of trauma. The mean number of dislocations was 5 per patient (range, 1 to 11). Patients with no history of trauma, with multidirectional atraumatic instability or without a finding of Bankart-type lesions were excluded. Patients were followed up routinely at 3 weeks, 6 weeks, 3 months, and 6 months postoperatively and at final follow up.

Using the National Centre for Biotechnology Information PubMed database relevant studies reporting results of the “purse string” technique were identified.

## RESULTS

Patients were followed up in the clinic for a minimum of 6 (range 6-24) months until discharged. Median follow up since surgery is 4.3 (range 1 to 9) years. Almost all patients (97%) returned to their sporting and leisure activities and all professional athletes went back to the same sport.

Overall 9 (4.7%) patients experienced recurrent instability symptoms at a median of 24 (range 6 to 36) months post-surgery. Three patients (1.6%) had recurrent subluxation and apprehension that was treated with physiotherapy. This happened at median of 21 (range 6- 26) months and in one of them, that was a professional athlete, occurred during a martial arts tournament when hit by another athlete. Six patients (3.1%) had recurrent dislocation requiring further surgery. Four of them sustained a new sports traumatic injury. Only one patient needed a Latarjet procedure and the rest were treated successfully with revision “purse-string” technique.

During literature research 1 paper was identified from a UK centre publishing similar results to ours. From 114 consecutive patients with anterior instability and a Bankart lesion treated with “purse string” failure rate was 6.1% at 4 years follow-up.

## DISCUSSION

Over the last years arthroscopic surgery has made significant advances both in materials and techniques. This has led to similar long term results between open and arthroscopic surgery for anterior shoulder instability [12, 13].

When using traditional arthroscopic techniques to stabilize the anterior labrum the number of suture anchors is reported as one of the most important factors affecting outcome. These techniques rely on using as many anchors as possible to “spot weld” the detached anterior labrum to the glenoid. As a result more “spots” lead to stronger repair and less than 2 anchors can cause failure [5, 14, 15].

The “Purse-String” technique (PST) is a different method to simultaneously address the Bankart lesion and anterior capsule laxity, using a double-loaded single suture anchor. The purpose of the PST is to repair the detached labrum, recreate the anterior bumper and shift the anterior- inferior capsule superiorly. These 3 repairs mimic the open vertical-apical suture technique [7] with the difference that in PST both Bankart repair and capsular shift are achieved on the glenoid side by extending the distances of suture limbs between 6- and 2-o’clock position [9]. With the PST the anchor is less important than the “purse- string” sutures that create approximation of the anterior capsule, reduce laxity and improve proprioception. The role of the anchor at the 4 o’clock position in PST is to ensure that the capsule is shifted superiorly and the anterior bumper attaches to the glenoid. The only setback is that rehabilitation has to be conservative and movement is restricted for the first six weeks of healing [9].

The senior author’s (O.L.) previous experience since 1998 that was published in 2006 [8] and 2007 [9] indicated very low recurrence rates of less than 6%. This continues to be evident in our latest results with a failure rate in the form of redislocation or instability symptoms of 4.7% (9 patients from 193). What is more important is that in 4 out of 9 patients recurrence was the result of a new violent incident (fall, motorcycle crush and martial arts injury).

One more centre in the UK uses the PST and has published similar results as ours. Failure rate for their series of 114 patients was 6.1% which is comparable to our 4.7%. These results are similar to other open and arthroscopic methods. In a recent review of 26 studies (1,781 patients) at mean of 11 years clinical follow-up recurrence of instability after arthroscopic techniques was 11% and after open 8% [13]. (Table **1**).

The “purse- string” suture, without an anchor in the glenoid, has also been used for arthroscopic rotator interval closure in symptomatic inferior shoulder instability. For this technique a single suture is passed from the superior glenohumeral ligament at the level of biceps tendon to the middle glenohumeral ligament overlying the subscapularis. This technique has been used to improve symptoms in multidirectional instability patients with more prominent antero- inferior instability symptoms. In 20 patients, at 2 years follow up, symptoms significantly improved and no re-dislocations were recorded [16].

## CONCLUSION

The “purse-string” technique has been shown to be at least as effective for the treatment of anterior shoulder instability, with similar failure rates and high patient satisfaction. At the same time it has several advantages over multiple anchor “spot-welding” technique (SWT). The most obvious is cost since one third of anchors are used. At the same time PST is faster than SWT for the same reason. PST requires only one anterior portal and one cannula and is less traumatic to the anterior soft tissues. Finally, in case of failure the fact that only one anchor has been inserted makes revision simpler leaving space for revision anchors to be inserted [8]. The “purse- string” suture can also be used in the very complex group of patients with multidirectional instability after careful selection and knowledge of the technique can lead to successful results in this difficult to treat condition [16].

## Figures and Tables

**Fig. (1):**
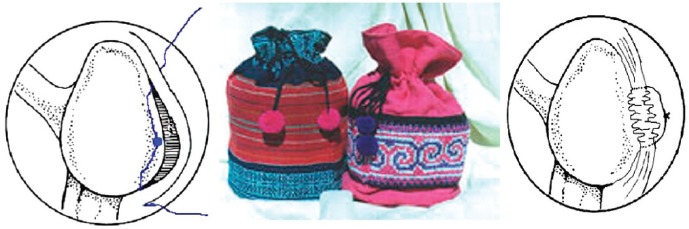
The suture through the capsulolabral complex tightens the tissue in the same manner as the drawstring of a purse [8].

**Fig. (2):**
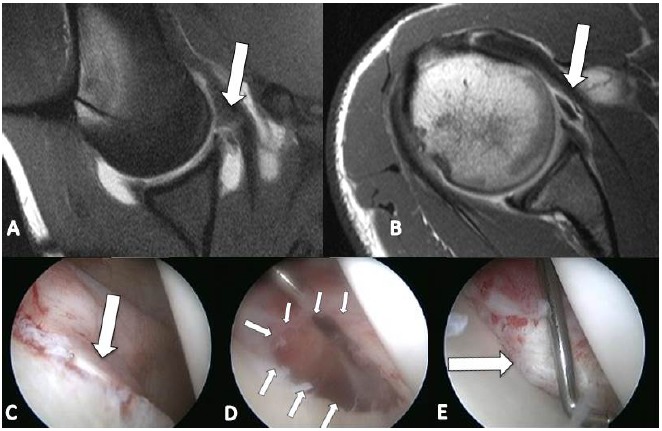
A,B: MR Arthrogram showing soft tissue Bankart lesion (white arrow); C: arthroscopic view (white arrow);D: Post liberation (multiple arrows); E Final repair (white arrow).

**Table 1:** Relevant papers in literature.

**Author**	**Year**	**Hospital**	**Patients**	**Recurrence (%)**	**Follow up (years)**	**Notes**
Levy O^8^	2006	Reading Shoulder Unit, Royal Berkshire Hospital, Reading, England	66	4.5	2	Original technique described
Levy O^9^	2007	Reading Shoulder Unit, Royal Berkshire Hospital, Reading, England	36	5.4	3	First long term results
Witney-Lagen C^10^	2014	Dewsbury and District Hospital, Halifax, W. Yorks, UK	114	6.1	4	PST results from another UK Hospital
Moon YL^16^	2011	Chosun University Hospital, Gwangju, South Korea	20	0.0	2	“Purse-string” suture to close rotator interval

## LIST OF ABBREVIATIONS

PST: = “purse-string” technique
SWT: = “spot-welding” technique
